# Study of impacts of brickkiln emanations on soil quality of agriculture lands in selected areas of District Bhimber, Azad Jammu and Kashmir, Pakistan

**DOI:** 10.1371/journal.pone.0258438

**Published:** 2022-02-11

**Authors:** Muhammad Ishtiaq, Iqbal Hussain, Khizar Hayat Bhatti, Mehwish Maqbool, Khawaja Shafique Ahmed, Muhammad Ajaib, Amin ullah Shah, Waheeda Mushtaq, Tanveer Hussain, Abdul Ghani, Humaira Khanum, Muhammad Waqas Mazhar, Mubashir Mazhar, Tauqeer Sardar, Omaima Nasif, Mohammad Javed Ansari, Peter Ondrisik

**Affiliations:** 1 Department of Botany, Mirpur University of Science & Technology (MUST), Mirpur, Azad Jammu and Kashmir, Pakistan; 2 Department of Botany, Government College University Faisalabad, Faisalabad, Pakistan; 3 Department of Botany, University of Gujrat, Gujrat City, Pakistan; 4 Department of Botany, University of Poonch, Rawalakot, Azad Jammu and Kashmir, Pakistan; 5 Department of Botany, Sargodha University, Sargodha, Pakistan; 6 Department of Physiology, College of Medicine and King Khalid University Hospital, King Saud University, Medical City, Riyadh, Saudi Arabia; 7 Department of Botany, Hindu College Moradabad (Mahatma Jyotiba Phule Rohilkhand University Bareilly), Moradabad, India; 8 Department of Environmental Sciences and Biology, Slovak Agricultural University, Nitra, Slovakia; Ghazi University, PAKISTAN

## Abstract

The pollution is hot issue of current era in world and the current study was carried to explore impacts of brickkilns’ emanations on physiochemical properties of agricultural lands from District Bhimber of Azad Jammu and Kashmir (AJK) Pakistan. In this research, various edaphic characteristics: pH, soil organic matter, organic carbon, water holding capacity, cation exchange capacity and heavy metal contamination in soils nearby of brickkilns were determined. The pH of soil ranged from 5.55 to 7.50, soil organic matter was 0.35–0.90% and organic carbon content was 0.65–1.40%. The water holding capacity ranged from 2.10 to 3.20 mgL^-1^ and carbon exchange capacity was 1250 to 4202 meq/100g. The contamination profile of heavy metal depicted that Pb showed highest conc. 0.065 mg/g followed by Co (0.053 mg/g) and Ni with 0.52 mg/g in the soil. Pb and Cr had high conc. in soil samples around brickkilns due to burning of coal and rubber tyres as fuel. The conc. of sulphate and nitrate ranged from 0.90±0.50 mol L^-1^ to 4.25±0.65 mol L^-1^ and 2.30±0.50 mol L^-1^ to 6.55±0.25 mol L^-1^, respectively. The fertility of agriculture lands was depicted that edaphic properties were directly related while nutritive features were inversely commensurate to distance from brickkilns. The research proved that emanations of brickkilns causes severe impact on quality of agriculture land, plant growth and its yield. Hence, reclamation measures should be taken to mitigate and/or eradicate nuisance of brickkilns emanations by using environmental friendly strategies.

## Introduction

Environmental pollution in urban and industrialized areas is becoming key issue in many developing countries of world and becoming serious threat accelerated and panic due anthropogenic activities and rampant rise of industrialization, motorization and urbanization [1]. The study of causes of increase in pollution is very pertinent because it impacts different aspects of human life. It causes loss in food quality as well quantity and primarily human health is directly disturbed. Exponential rise in population demands more houses and infrastructures to cope necessities of humanity. High demand of bricks has triggered to initiate start-up of many brickkilns in many cities and villages of the country. The quality of air has deteriorated due to increase in particulate matter conc., toxic emissions like SO_2_ and nitrogen oxides (NO_x_) from brickkilns’ emanations [2, 3]. Increased pollutants of different heavy metals along with sulfur dioxide, nitrogen compounds in air has unpropitious and lethal effects on agricultural soils and crop yield which may enter food web and become toxic for human health [4]. The increase in air pollution due to brickkiln emanations is prevalent cause of arable quality loss and this is being continuously in vague since last many decades.

In developing countries like Pakistan, there is already scarcity of paved houses and hitherto majority people of rural areas live in mud-houses. In current scenario, in pursuance of rapid economic growth and development need of good infrastructure has key priorities in Pakistan while for protection of environment has not been given such pivotal significance. The haphazardly setup of industries and brickkilns has lead deterioration of natural resources. As consequence, pollution is tremendously increased maneuvering serious climatic changes. Pakistan has 8–18% brickkilns of world and mostly brick furnaces are being operated in different populous cities, towns and villages [5]. The overwhelming startup of kilns near arable lands has created degradation of land soil quality which has shed severe impacts on productivity of crops and food security. Hence, there is urgency for taking steps to restore land loss and fertility by mitigation of brickkiln pollution to regain good crop productivity with food security.

The key parameter in increase of brickkiln pollution in country is their old-model structure and use of substandard and cheap fuel i.e. coal, rubber tyres, crude oil etc which determines toxicity level [5]. The ignition of low quality fuel produces fine soot particles, hydrocarbons, CO, Fluorides, SO_2_, NO_x_ and other particulate matter which causes different respiratory infirmities and cancer [6–8]. The brickkiln emanations comprise of heavy metals like Cu, Co, Pb, Cr, Zn, Cd, Mn and Ni which are very injurious to health, if their conc. is above WHO safety limits [9]. The high conc. of heavy metals also have severe impacts on plant growth and fruit and grain yield [10]. These metals become part of plant body penetrating through root absorption and then move in food chain when eaten by animals and human which causes many health threats to man and other living organisms [11–15]. The studies reveal that micronutrients like Co, Se, Zi, Cu and Mo are essential for plants in optimum form but other metals such as Ni, Cd, Hg and Cr are unnecessary and mostly toxic if their conc. rise above than safe values [16, 17]. It is recommendation of WHO that prior to eating, food from plants or other sources must be analyzed for heavy metal conc., bacterial and fungal contamination for secure health. The food with high conc. of heavy metals is serious issue of the time. It is reported that “itching disease” out-broke in Japan due to high level of Cd in rice food and it is also reason of osteomalacia disease. Similarly, high content of Hg taken through fish consumption may cause Minamata infirmity, renal disorders and cancer [18–20].

**Fig 1:**
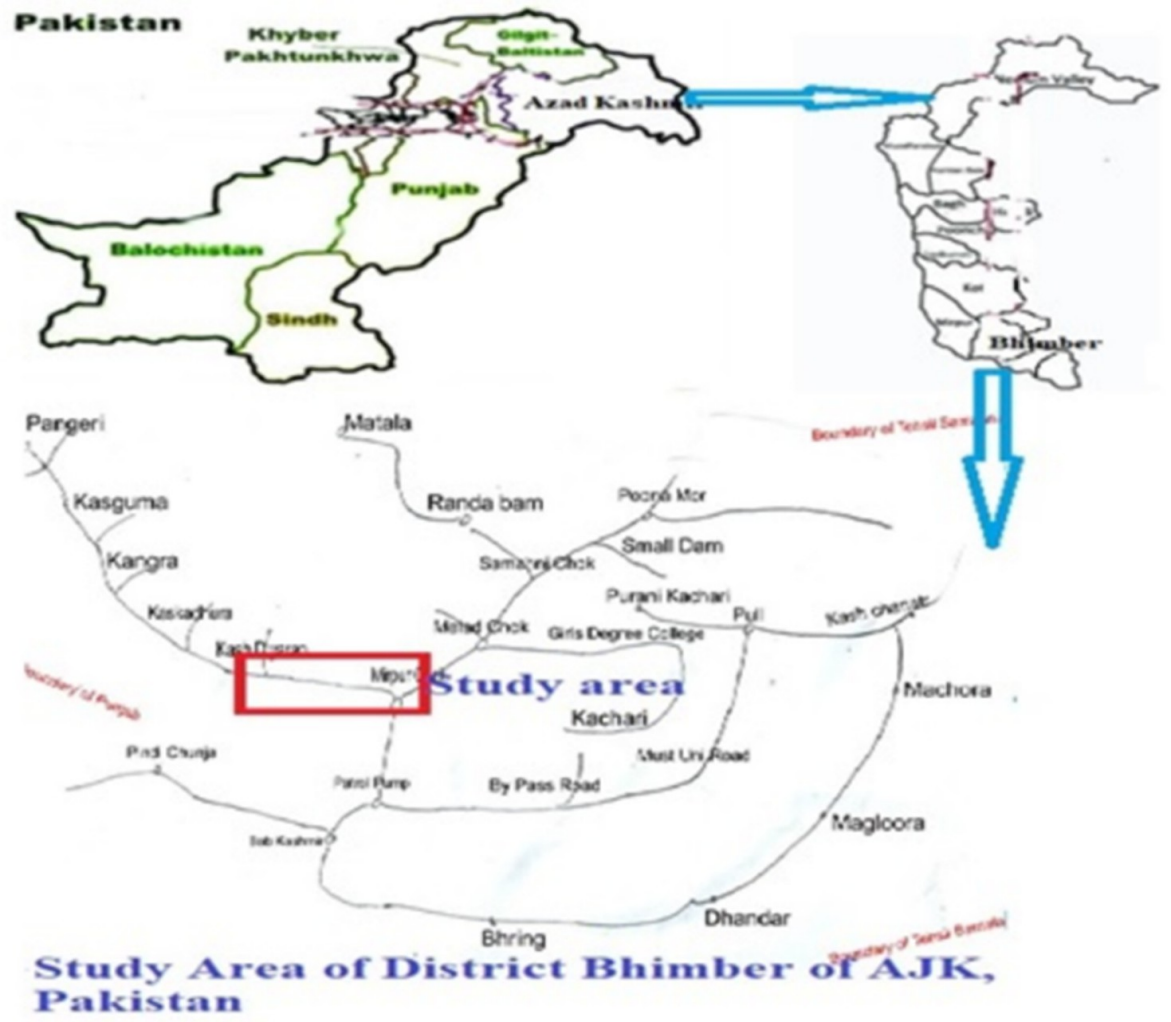
Map of study area (Brickkilns site-Pooteeh) of District Bhimber Azad Jammu and Kashmir, Pakistan.

The current study was planned to explore effects of brickkiln emissions on nearby arable soils from Pooteeh area of District Bhimber, AJK Pakistan. Geographically the study area is located between 32–48 to 33–34^⸰^ latitude and 73–55 to 74–45^⸰^ longitude as shown in Fig 1 and described by previous researchers [21]. The study area is mostly arid and scrubby vegetation is present. The commonly found wild flora is *Acacia nilotica*, *Ziziphus jujuba* Mill., *Butea monosperma* (Lam.) Taub., *Adhatoda vesica* Nees. and *Dalbergia sissoo* Roxb. Among crops Maize, Millet and Wheat are cultivated in the area, and its growth and yield depends on soil, organic matter and seasonal rain fall. The people use traditional farming system of cultivation of crops without use of latest ploughing and sowing techniques. Mostly people are interlinked with kiln industry for earing livelihood by baking, shifting and loading on vehicles. Hence, this area has rich biocultural diversity and makes this area interesting for research.

Currently there are many brickkilns working in District Bhimber but neither standard managing rules for making specific structure of kilns are formulated by Government nor implemented by the owners and same is plethora of raw fuel use. The most brickkilns prevalently use cheap and substandard fueling material like coal, rubber tyres whose fallout is creating direct threats to health of children, women and men who are connected with it for labour and also deteriorating arable land quality of vicinity.

This is first time study conducted on analysis of impacts of brickkiln emanations on arable soil quality from District Bhimber of Azad Jammu and Kashmir, Pakistan. The hypothesis of research was based on: “whether common use of coal and rubber tyres as fuel source and classical design of brickkilns is key cause of rapid increase of pollution and deterioration of agriculture lands”?? The objectives of research were to (i) analyze physiochemical properties of soil, and (ii) determine conc. of heavy metals (Cu, Co, Pb, Cr, Zn, Cd, Mn and Ni) in soil near to brickkilns and its impact on soil fertility.

## Materials and methodologies

### Determination of sampling sites

The sampling of brickkiln sites were selected from agriculture farmlands present in vicinity of brickkilns from “Poteehy area” of District Bhimber, AJK. The most of brickkilns are in center of crop lands. The sampling sites were selected based on four distance (100m, 200m, 300m and 400m) and four directions from kilns. The common or classical form of brickkiln chimneys were column shaped or vertical shaft form. There are no other models being hitherto employed in chimney models in the study area. So, study is based on only vertical shaft or column shaped kilns of District Bhimber of AJK. The wind direction and speed was measured by anemometer model KM 908.

### Collection of soil samples

Soil samples in triplicate from depth of 0–50 cm from distance of (*R* = 100m, 200m, 300m and 400m) and from four sides (*D*) were collected. The samples were packed, tagged systematically and brought to laboratory for further analysis. The labeling each sample was based code *RD* where *R* is distance and *R* is direction from kilns. The samples were cleaned from external substances, air dried and oven dried at 120 °C for two days. The dried samples were sieved using sieving apparatus (10>0.075mm pores). The separated samples with > 2mm soils were ground and preserved in desiccator for next analysis using protocol of El-Balkhi et al., 2009 with some modifications [22].

### Analysis of soil samples

A purified soil sample (50g) was taken in 250 mL conical flask and 50 mL of dist. water added in it. The mixture was shaken for 10 min and left on bench for 30 min and filtered using Whatman paper No.42. The turbid samples were centrifuged at 3000 rpm for five minutes and processed further. In soil physiochemical experiment, 100 mL of dist. water was added to 10g soil which was poured in a flask and kept stagnant for 2–3 hrs for future use using method of El-Balkhi et al., 2009 with some alterations [22].

### Physical parameters of soil samples

In this analysis, 10gm of soil was taken in funnel and 100mL of water was admixed and capped with glass stopper. To check soil water holding capacity (WHC), soil samples were kept on table for 2–4 hrs so that soil can absorb maximum water. The soil texture was calculated by using standard international pippete protocol [23]. The pH of soil was determined by using double distilled water in ratio of (soil: water) 1:2.5 (w/v) following procedure of Chauhan, (2010) [23]. The organic carbon content (OCC) was explored following methodology of Walkley and Blake (1947) [24]. The soil organic matter (SOM) was determined by applying standard protocol following procedure of titration technique as per method of Nelson and Sommers (1996) [25]. To determine organic matter (OM), 1.724 conversion factor was applied using protocol of Alam *et al*., (2013) with some changes [26, 27].

### Soil fertility of samples

To calculate soil fertility quantity of sulphate and nitrate in soil samples was explored. Firstly, a reagent was prepared by admixing 15mL of conc. HCl with 50mL of dist. water and 50mL of 95% isopropanol. About 37.5g of NaCl was dissolved in it and 25mL of glycerol was mixed in the solution [28]. Standard solution of Na_2_SO_4_ was prepared by dissolving 1.479 g in distilled water. For determination of fertility (sulphate), analysis of sulphate standard solution, conditional reagent and sample solution was conducted at 420 nm wavelength using Genesys UV-180 visible spectrophotometer following protocol of Gupta, (2000) [27]. First standard solution curve was obtained and then conc. of sulphate was determined in different soil sample solutions. The calorimetric method was used to determine conc. of nitrate in the soil samples. In this process, 0.01M conc. solution, MgCO_3_, Ca(OH)_2_ solutions were used and clear sample extract was obtained. In the procedure, H_2_SO_4_ reacts with nitrate contents and produces nitric acid which makes changes of nitration in 2,4-phenoldisulphonic acid in its dry condition state. The conc. of color will be used for determining of conc. of nitrate in samples using spectrophotometer absorbance at 415 nm. First standard curve will be prepared using different conc. of NO_3_ solutions and conc. of nitrate in soils samples will be carried out following protocol of Yaseen *et al*., 2015 [29]. All experimental trials were run in triplicate form to keep good reliability and authenticity of analysis.

### Heavy metal conc. in soil samples

The conc. of heavy metal was determined following protocol of Yaseen et al., (2015) and Wuana and Okieimen, (2011) [29, 30]. In analysis, one gram of processed soil was dissolved in 15 mL of aqua regia (HNO_3_/HCl, 1:3, v/v) in flask and kept for 24 hrs. The flask was heated at 50°C for 30 minutes with gradual rise of temperature upto 120°C and kept for 2 hours. Then flask cooled and 10mL of 0.25 M HNO_3_ was added in it. The solution was filtered using Whatman paper No. 542 -and filtrate’s volume was raised to 50mL using 0.25 M nitric acid [31–33]. The prepared soil samples were analyzed using atomic absorption spectrophotometer (AAS) model 700, China) and analysis was conducted in triplet and average value was determined. The soil nature or texture was calculated using international pipette method (IPM) and soil classes were separated and named using percentage of sand, silt and clay in the sample as per texture classification system of USDA. The collected data was analyzed using relevant statistical tools.

#### Statistical analysis

The collected data was tabulated and analyzed through ANOVA using SPSS software version 26.0. The results were presented in tables describing significance level for the different samples.

## Results and discussion

The current research was planned to determine toxic impacts of brickkiln fallouts on agriculture soils occurring near brickkilns of selected sites of District Bhimber of AJK. The emanations of kilns contain different heavy metals whose high conc. level exert severe impacts on physiochemical properties of agriculture soils and grown crops.

The soil texture analysis depicted that mostly soil was ‘silt loamy’ around the brickkilns. This type of soil generally has optimum conc. of organic matter (OM) but high contents of pollutants of kilns have detrimental impacts on it and it coincides previous published research works [34, 35]. It was found that soil samples contained moderate to high quantity of soil organic matter (SOM) ranging from 0.35–0.90% depending on distance and direction from brickkilns (Table 1). These research findings are similar with past works of Achakzai *et al*., [33] and Tammeorg et al., (2014) who stated that around kilns SOM quantity was variable with distance and direction [34, 36]. To find soil quality, organic carbon content (OCC) of samples was calculated which ranged from 0.65–1.40% (Table 1). It was found that OCC was less 0.0880 at *R* = 100 in nearby soils of brickkilns and when distance was increased conc. of OCC was raised making soil the least fertile. The organic carbon content of arable soil is very important property of land has significant impact on crop growth and yield. Hence, deterioration of OCC due to kilns’ pollution severely impacts the fertility of arable land.

**Table 1:** Impacts of distance and direction of brickkiln on soil, organic matter and organic carbon content from District Bhimber of Azad Jammu and Kashmir, Pakistan.

Distance	Direction	Sand%	Silt%	Clay%	Texture Class	Soil Organic Matter %	Organic carbon content %	pH	Water holding capacity
100m	East	9.55	74.95	15.50	Silt-loam	0.48	0.73	5.75±0.55	2.90
	West	8.90	71.85	19.25	Silt-loam	0.64	0.82	6.05±0.22	2.75
	North	7.45	74.05	18.50	Silt-loam	0.46	0.73	5.60±0.10	3.05
	South	12.25	66.50	21.25	Silt-loam	0.71	0.67	6.90±0.25	2.85
200m	East	10.25	70.75	19.00	Silt-loam	0.39	0.90	6.25±0.15	3.70
	West	11.50	69.00	21.50	Silt-loam	0.56	0.83	6.50±0.25	3.90
	North	10.55	69.50	19.50	Silt-loam	0.55	0.92	7.40±0.25	3.95
	South	22.00	61.45	16.55	Silt-sand	0.72	0.78	8.55±0.56	3.75
300 m	East	17.50	65.00	17.50	Silt-sand	0.56	1.20	6.85±0.25	4.05
	West	19.85	63.90	14.25	Silt-loam	0.62	0.93	6.90±0.60	4.50
	North	18.55	63.90	17.55	Silt-loam	0.70	1.14	8.25±0.55	4.72
	South	17.45	64.05	18.50	Silt-sand	0.81	1.09	7.20±0.65	4.90
400 m	East	22.50	57.50	20.00	Silt-loam	0.87	1.30	7.90±0.10	5.45
	West	21.80	56.70	21.50	Silt-loam	0.92	1.45	8.20±0.05	6.75
	North	17.40	62375	20.85	Silt-sand	0.86	1.55	8.75±0.55	5.65
	South	25.75	51.25	23.00	Silt-loam	0.81	1.90	8.55±0.55	5.25

The high temperature near the sink causes breakdown of soil organic matter (SOM) in form of release of CO and CO_2_. It was found that SOM quantity was comparatively less in near land’s samples of brickkilns than samples taken from far distance. The pH of soils near to kilns was low (5.75±0.55 for *R* = 100m) because of SO_2_ release and H_2_SO_4_ production when Sulphur dioxide mixes with water and this creates acidity with low pH [34]. It is known that heavy metal conc. in soil is dependent on many factors such as pH, organic carbon content (OCC), soil organic matter (SOM) and cation exchange capacity (CEC). In plants metal absorption is correlated with factors such as metal solutions, labile phase, absorption equilibrium and solid-liquid phase stability around the plants’ roots [37].

Soil with better values of OCC is considered good for structure, aeration and water penetration property. A strong correlation between OCC and water holding capacity (WHC) of the land was found which had strong impact on availability soil nutrients to the plants. OCC determines quality of soil and it improves soil pores, aeration, soil structure and water holding capacity (WHC). This generally assists in nutrients supply to plants for good growth and if any soil has more than 0.80% OCC, it is known as good quality soil, some researcher reported that if OCC more than 0.75% are also considered as good soil for crop cultivation and these results are also proved by different previous research works [34, 38]. The results depicted the lowest value (0.65%) of OCC was found in the sample collected at distance of 100m from southern side of kiln while the highest (1.40%) was indicated in sample collected from south-side at distance of 200m from sink. This variation in OCC conc. in different directions and distances may be due to factors that soil surface is removed during raw brick preparation or ratio of SOM in the samples. The quantity of soil OCC may be correlated with soil texture as clay-loam soil has better outcomes than clay-sandy soil because former holds OCC by chelation and similar findings were reported by previous researcher [34]. The less conc. of OCC and SOM at nearby samples or soil from sink or brickkiln may be due to high conc. of pollutants which severely impact these two parameters of soil (Table 1) and these our results are congruent with results of Bisht and Neupane, (2015) [37]. The other factor which causes variation in OCC and SOM is application of chemical fertilizers in agriculture lands for crop yield increase by native farmers without prior edaphic analysis which makes it irrational approach.

Generally, pH of soil represents ecological and edaphalogical health of soil and agriculture soils. The analysis depicted that pH of soil samples ranged from 5.55 to 7.55 (Table 1) and its range increased when moving away from sink (kilns) and direction of pollution source. These findings are congruent with past works of Bisht and Neupane, (2015) who stated that distance and direction from kilns is relevant to pollution in arable lands [37]. The pH of land does have direct impact on types and population density of soil microorganisms (SMOs) and provision of nutrients to plants, so health of plant and yield is effected by pH of agricultural soil. The optimum availability of micronutrients (metal elements) was found in pH range of 6.30 to 7.50 (Table 1) and our findings were coincidence with previously published works [34, 38, 39]. The presence and absorption of elements is dependent on pH of soil and particularly Zn, Cu and Ni are significantly correlated with it [40]. The occurrence and availability metals is related with cation exchange capacity (CEC) of soil except Zn element. The conc. of Zn and Pb metals is directly linked with amount of SOM and OCC in arable soils (Table 1). SOM has also significant relationship with metals storage, availability to crops because SOM makes chelates with micronutrients which assist in transport supply [41].

It is also known that if pH of soils is acidic then mobility of micronutrients: lead (Pb), cadmium (Cd), chromium (Cr), copper (Cu), nickel (Ni), cobalt (Co), molybdenum (Mo) and zinc (Zn) is increased while for alkaline lands it is reversed. The pH of soil is indicator of plant growth and if pH is low then it retards absorption of metals which impedes plant growth and yield. It was found that pH near (100m distance) to brickkiln was 5.55 for eastward and 6.25 for northside sample which depicted that near to sink there is low or acidic pH, so it depicts the impact of emissions of brickkilns (Table 2). The low pH on eastside may be due to wind direction from west to east which carries fallouts towards eastward. These results are coincident with work of Chauhan (2010); Gupta, (2000) who descried that near kilns pH was low due to excessive pollutant fallouts [23, 27].

**Table 2:** Relationship between available heavy metal conc., soils characteristics and pH from brick kiln area of District Bhimber AJK, Pakistan.

Name of heavy metals	pH	OCC	SOM	CEC	Clay
Cu	–0.0255*	0.3485*	0.1795**	0.1810	0.0245
Co	–0.1325	0.2650**	0.5908*	0.2590*	0.4420*
Zn	–0.2305	0.1425	0.3955**	0.1250**	0.0255
Pb	–0.2055**	0.2455**	0.5390	0.2680	0.1450*
Cr	–0.5240	0.3070	0.5390	0.4502	0.0205
Ni	–0.3055	0.2420	0.1985**	0.3210**	0.2595*
Cd	–0.1035*	0.2095**	0.3590	0.3650	0.0355
Mn	–0.3150	0.3225	0.4380*	0.2505	0.3130*

* and ** means significant at 5%, and 1% level, respectively.

The water absorption capacity (WAC) is important parameter for determining quality of soil. In analysis, it was found that WHC of all samples ranged from 2.10 to 3.20 mgL^-1^ (Table 3). It was observed that soil samples taken from southern side have better WHC than other samples. There was not considerable difference of WHC for distance from the source except the sample taken from 200m-southwise which had 3.20 mgL^-1^ that might be due to difference of structure and texture of soil (Table 1). These results of research are some similar with previous works of Debnath, *et al*., (2012); Bisht and Sanjila, (2015) who demonstrated that WHC is linked with soil texture and structure [37, 40]. It is- valuable to state that if WHC is less then soil has deep water table and not appropriate for growth of crops. It was explored that soil near to brickkilns has low WHC that may be due to more heat or high temperature and movement of workers and vehicles makes the soil hard, so there is less chance of water infiltration and result is lowering of ground water table, ultimately making that soil unfertile and barren. Continuous heating and anthropogenic activities or other biotic and abiotic interferences on soil may cause change in soil structure and texture leading toward less appropriate for any growth of plant or preferably for agriculture cultivation and productivity and similar findings had been cited by Gupta, 2000 [27].

**Table 3:** Relationship between total heavy metal conc., soils characteristics and pH from brick kiln area of District Bhimber AJK, Pakistan.

Name of heavy metals	pH	OCC	SOM	CEC	Clay
Cu	0.3170*	0.1720	0.2027*	0.4290*	0.4056**
Co	0.3050**	0.1685	0.0870	0.3710**	0.1808
Zn	0.0275	0.5070*	0.3677*	0.4250*	0.2069
Pb	-0.3105	0.3290**	0.2690	0.3505	0.5047*
Cr	0.1895**	0.0880	0.2680	0.2420*	0.2258
Ni	0.1277	0.5010*	0.4950*	0.4552*	0.3565**
Cd	-0.3050*	0.2155**	0.2251*	0.3580**	0.5040*
Mn	0.0590	-0.0370	-0.0255	0.1655	0.2205*

* and ** means significant at 5%, and 1% level, respectively.

A positive correlation was determined among pH, cation exchange capacity (CEC), SOM, OCC and soil texture type (Table 2). The CEC values of analyzed soil samples ranged for 1250 to 4202 and—conc. of Zi and Ni metals depicted significant correlationship with CEC. Similarly, SOM has ability to make chelate with metals and there was strong correlationship with conc. of heavy metals and SOM amount in the soil (Tables 2 and 3). In the heavy metals analysis, a significant correlation of SOM with conc. Zn, Ni, Cu and Mn was found while availability of heavy metals was interlinked with OCC in the samples (Table 3). These findings are similar with previous works of Johnson and Petras, (1998) and Bradl, (2004) who stated that SOM and OCC had paramount impact on conc. of heavy metals in soil samples collected from vicinity of brickkilns [38, 39]. Similarly, Sikder et al., (2016) discovered that SOM and metals availability from soil was dependent on SOM and OCC [41].

It was found that conc. of heavy metals like Co, N, Mn and Pb was higher in dust or upper layers of soils and its conc. gradually becomes less with increase in soil depth (Table 4). Pb ranked 1^st^ with 0.065 mg/g followed by Co (0.053 mg/g) and Ni with 0.52 mg/g. It was found that high conc. of heavy metals was determined in top soil and near to brickkilns than subsoils or deep soils. Similar reports had been published in the literature about presence of more conc. of metals in upper and near to brickkiln soil layers by previous researchers [42, 43].

**Table 4:** Impact of brickkiln direction and distance on conc. of heavy metals in soil from Bhimber AJK, Pakistan (elements (mg/g).

Direction	Distance	Cu	Co	Pb	Cr	Zn	Cd	Mn	Ni
East	100m	0.014±0.005	0.004±0.008	0.054±0.012	0.045±0.015	0.046±0.015	0.006±0.015	0.466±0.025	0.024±0.055
	200m	0.025±0.002	0.005±0.012	0.048±0.010	0.041±0.020	0.049±0.022	0.009±0.012	0.551±0.055	0.039±0.025
	300m	0.045±0.006	0.007±0.015	0.037±0.025	0.039±0.080	0.051±0.045	0.007±0.015	0.680±0.045	0.048±0.075
	400m	0.059±0.007	0.009±0.020	0.028±0.020	0.034±0.045	0.053±0.090	0.005±0.075	0.710±0.026	0.049±0.080
West	100m	0.012±0.003	0.005±0.002	0.052±0.015	0.049±0.025	0.048±0.025	0.005±0.025	0.442±0.075	0.025±0.050
	200m	0.020±0.001	0.006±0.005	0.040±0.020	0.040±0.015	0.046±0.040	0.006±0.010	0.585±0.025	0.038±0.005
	300m	0.040±0.005	0.006±0.025	0.041±0.035	0.038±0.050	0.051±0.035	0.003±0.085	0.677±0.010	0.049±0.070
	400m	0.050±0.004	0.008±0.002	0.055±0.055	0.028±0.055	0.055±0.055	0.004±0.060	0.690±0.045	0.047±0.050
North	100m	0.011±0.003	0.004±0.040	0.059±0.042	0.043±0.020	0.043±0.005	0.005±0.005	0.427±0.044	0.023±0.065
	200m	0.022±0.003	0.006±0.001	0.038±0.020	0.046±0.070	0.045±0.029	0.008±0.002	0.585±0.001	0.036±0.065
	300m	0.042±0.004	0.007±0.002	0.041±0.020	0.032±0.065	0.050±0.058	0.004±0.070	0.610±0.049	0.043±0.025
	400m	0.062±0.005	0.007±0.025	0.038±0.045	0.026±0.015	0.054±0.075	0.006±0.055	0.686±0.075	0.044±0.050
South	100m	0.018±0.002	0.004±0.050	0.057±0.035	0.049±0.045	0.048±0.025	0.007±0.075	0.462±0.095	0.026±0.051
	200m	0.021±0.005	0.006±0.025	0.042±0.050	0.037±0.090	0.047±0.020	0.006±0.010	0.590±0.050	0.049±0.015
	300m	0.043±0.001	0.005±0.090	0.032±0.020	0.035±0.040	0.046±0.085	0.008±0.025	0.670±0.055	0.047±0.020
	400m	0.055±0.002	0.005±0.065	0.061±0.055	0.022±0.035	0.051±0.050	0.004±0.050	0.758±0.025	0.045±0.088

It was determined that conc. of different heavy metals in soil was variable due to many biotic and abiotic factors impacting on it. It was found that conc. of Cu ranged from 0.011 to 0.062mg/g and it has positive correlation with distance and direction from brickkilns (Table 3). It was determined that soil samples collected from east and north side had more quantity of Cu, which might be due to wind direction and similarly distance of soil sample sites also had significant impact on its quantity. Key sources of Cu in soils is from fertilizers, industrial effluents, sewerage sludge and pesticides while here in these soil samples Cu may be due to kiln emanations. The critical value of conc. of copper in plants is between 20 to 100 mg/Kg while for soils safe limit is <36mg/kg. It was determined that upper surface of soil has less conc. of Cu and vice versa that was due to chelate formation and leaching with water along with SOM triggers it down to deep soils. These findings are similar with published works of Hussain, (2006) and Ishaq et al., (2010) [44, 45]. It was determined that conc. of Pb in different soil samples was variable and inversely linked with distance from brickkiln. Its conc. ranged between 0.028 to 0.054 mg/g in analysis (Table 4). The key cause of Cu was burning of rubber tyres in brickkilns and higher conc. is injurious for plant growth.

The conc. of other metal chromium (Cr)—is decreased d with increase of distance and depth of soil samples taken from vicinity of kilns This metal leaches down with water from acid rain and then accumulates in soil and enters in plants’ systems through roots by water movement (Table 4). This research finding is confirmed by the previous findings of Sikder *et al*., (2016) and Hussain, (2006) [41, 44]. The analysis depicted that conc. for Cr was 0.032 to 0062 mg/g (Table 4). It was explored that conc. of all heavy metals (Cu, Co, Mn, Ni, Cd and Zn) was high near to the source of brickkiln and their quantity decreased in far distance of soil samples from sink (Table 4) and similar results had been cited in past workers [46, 47]. The high conc. of Pb in soil samples is due to burning of coal and particularly rubber tyres for heating chimney. The other factor may be traffic movement on the vicinity of the sink but this input is very minute or nominal [46]. There is congruence found between the results of this study and previous work of Achakzai et al., (2017) [33].

The Co metal is also known as trace element and it is essential for regular functioning of plant. In this study, albeit Co conc. was below the critical values in soil and plants analytes (Tables 2–4) yet its study is very essential for proper analysis of soil and its correlation with kiln emanations. The Co is important for plant stem and leaf maturity while it is inevitable for healthy bud formation. These findings are congruent with work Myers and Davidson (2000) [43]. In the analysis, conc. of Zn in soil was within safe limits and this metal is important for chlorophyll formation and conversion mechanism of starch to glucose and vice versa. These results of the research are consistent with previous reports of Sikder et al., (2016) [41]. The high conc. of Pb in the soil was near brickkiln and towards eastern side and that is proved that it comes from fallout of chimney and move with direction of wind on ground. The conc. of lead (Pb) was 0.022 to 0.046 mg/g and it is known as toxic for living organisms and particularly for human being because it causes cancer. The continuous exposure to lead or eating with food may develop infertility or miscarriage of neonates in women [48].

It is explored that chimney emissions are key cause of rise of Cr. in soil and its conc. was higher near the sink (brickkilns) and dependent on direction due to wind [49, 50]. The metal is harmful for seed germination and it also effects growth of plants. When these contaminated parts of plants are eaten by animal or consumed by man had severe toxicity due to excessive conc. of heavy metals beyond safety limits causing different infirmities i.e. asthma, lung cancer, hypersensitivity and nasal cancer [18, 34]. The Cr is world’s known toxic agent and its common sources are tanneries, sewerage water of industries, brickkilns and domestic sources. Its conc. <5mg/Kg is safe while above it upto 30mg/Kg are recognized as critical causing damage to plants and human being and beyond 100mg/kg are very toxic as per WHO standards. When Cr. is taken/absorbed by human due to eating different contaminated foods it also causes skin itches, stomach ulcers, renal issues and cancer of lungs [49, 50]. The current research proves that soil and plants samples have different conc. of this heavy metal but these are within safe limits but if precautionary measures are not taken on priority, it may lead towards toxicity. Ni metal is commonly found in soil which is within safety limits (0.049±0.080 mg/g) and if it is continuously increased due to kilns’ emanations that will go beyond critical (safety) limits then it may cause itching or “Ni-itching” and cancer in human. The trend of Ni occurrence soil and plants was more in samples collected from near of brickkilns. The mode of distribution of the metal was through water movement.

Cd is toxic metal which causes health problems for man and it enters in food chain through soil and fertilizers, sewage, fuel burning, lead and zinc mine industries plants then goes into milk, meat and human body which enters in human causing many severe acute and chronic infirmities [43, 50]. The Cd critical value is 3-5mg/Kg and presence of Cd in plants indicates its movement from soil and other sink sources into plants and ultimately reach in human body when eaten. Another metal called manganese (Mn) is called essential element for plant growth by controlling enzymes but its high limits are toxic for living organisms. Mn safe limit is 300-350mg/Kg and its conc. in all soil and plant samples were within safe limits. If taken by man due to eating of contaminated plant parts, it causes muscle spasm, trembling in waling and tremors and similar findings have been reported in previous research workers [27, 50].

The fertility of soil becomes variable (less fertile) due to contamination of heavy metal concentration with different forms depending on its distance and wind direction. The conc. of pollutants was higher in far land areas in wind flowing direction, preferable in east to west and vice versa whereas on north and south side no symptoms of heavy metals were found in far distance. For analysis of soil fertility, conc. of sulphate was determined and its conc. was found least (0.90±0.50 mol L^-1^) at distance of 100m in north side while with increase in distance from kilns its conc. increased (4.25±0.65 mol L^-1^) at sampling site of 400m in west side (Table 5). The analysis depicted that conc. of nitrate fluctuates with distance and direction from pollution source (brickkilns). Other physiochemical properties of soil and conc. of heavy also have detrimental impact of fertility parameter (conc. of nitrate) and directly it influences the crop growth and yield culminating into indirect on economy and human life [49].

**Table 5:** Conc. of sulphate (mol L^-1^) in different soil samples taken from different distances from brick kilns furnaces from District Bhimber of AJK, Pakistan.

Direction	Distance			
	100m	200m	300m	400m
East	1.35±0.02	1.95±0.30	2.55±0.90	3.95±0.55
West	1.42±0.45	2.20±0.55	2.80±0.55	4.25±0.65
North	0.90±0.50	2.40±0.01	2.75±0.25	3.55±0.40
South	1.10±0.80	2.10±0.65	2.55±0.01	3.40±0.90

For determination fertility of arable soils around brickkilns, nitrate conc. in the analyzed samples was 2.30±0.50 mol L^-1^ at distance of 100m in southern side. While its quantity (conc.) were highest (6.55±0.25 mol L^-1)^ in east-ward sites at distance of 400m (Table 6). The conc. of sulphate and nitrates in soils samples indicates the impact of kiln emanations on fertility level of soils and usually it was found that soil fertility of samples of arable lands was reduced/ becoming low due to kilns’ emanations. The study proves that there is severe toxic impact of pollutants and emanations of brickkilns on soil fertility which has definite hazardous impeding effects on crops growth and yields. These studies are corroborates with previous researcher who carried similar works and our results are endorsing to those [37, 49, 50]. Hence, there is dare need of hour to devise mechanism to control pollution emanations from brickkilns by introducing latest kilns’ chimney structure and use of proper fuel which has the least or nonth toxic emissions. The green technology use may be introduced for reduction of pollution due to brickkilns emanations.

**Table 6:** Conc. of nitrate (mol L^-1^) in different soil samples taken from different distances from brick kilns furnaces from District Bhimber of AJK, Pakistan.

Direction	Distance			
	100m	200m	300m	400m
East	2.55±0.52	3.90±0.50	4.50±0.10	6.55±0.25
West	2.40±0.15	3.70±0.25	4.80±0.05	6.20±0.60
North	2.90±0.90	3.10±0.05	4.55±0.55	5.90±0.20
South	2.30±0.50	3.70±0.05	4.05±0.90	6.20±0.50

## Conclusion

The importance of current study is very vital because it provides analysis of core issues “pollution” due to brickkiln emanations on soils which are being used for cultivation of crops and its hazardous effects on human health. The significant impacts of brickkiln fallouts on properties of agriculture soils and its subsequent impacts on crops from District Bhimber of AJK has been presented. The physiochemical properties of agriculture land are modified and conc. of heavy metals also increased due to brickkiln pollution. The soils near to the sink were more severely affected by kilns’ emanations due to heavy metals pollutants. The distance of sink (kilns) and wind direction -influence quantity of pollutant emissions of brickkiln. Major cause of rise in heavy metals conc., particularly Cr and Pb in the area is due to burning of rubber tyres (used as fuel) in brickkilns and form of chimney structure. The study recommends to use latest and environment friendly techniques to mitigate the brickkiln pollution. The research recommends to use zigzag form of brickkiln chimney structure, impose bane on rubber tyres or other rubber byproducts to be used as fuel and prepare rules and regulations for mitigation of brickkiln pollution emission for safe and clean environment. It will assist in reclamation of arable soils for safe and secure food (grain) production from cultivation of different crops in the area. This will assist in mitigation of emergence of fatal chronic and acute diseases of human being.
