# Correction: Study of impacts of brickkiln emanations on soil quality of agriculture lands in selected areas of District Bhimber, Azad Jammu and Kashmir, Pakistan

**DOI:** 10.1371/journal.pone.0322230

**Published:** 2025-04-04

**Authors:** Muhammad Ishtiaq, Iqbal Hussain, Khizar Hayat Bhatti, Mehwish Maqbool, Khawaja Shafique Ahmed, Muhammad Ajaib, Amin ullah Shah, Waheeda Mushtaq, Tanveer Hussain, Abdul Ghani, Humaira Khanum, Muhammad Waqas Mazhar, Mubsher Mazher, Tauqeer Sardar, Omaima Nasif, Mohammad Javed Ansari, Peter Ondrisik

The thirteenth author’s name is spelled incorrectly. The correct name is: Mubsher Mazher.
